# Metachronous Gastric Metastasis of Hepatocellular Carcinoma Mimicking Early Gastric Cancer and Detected on Esophagogastroduodenoscopy: A Case Report and Literature Review

**DOI:** 10.3390/diagnostics16213602

**Published:** 2026-11-01

**Authors:** Jae-Hui Kim, Moonsik Kim, Jongmin Park, Jihoon Hong, Min Kyu Kang, Yu Rim Lee, Seong Woo Jeon, Hyung Jun Kwon, Nora Jee-Young Park, An Na Seo

**Affiliations:** 1 Department of Pathology, School of Medicine, Kyungpook National University, Daegu 41405, Republic of Korea; jhkimpath@knuh.kr (J.-H.K.); teiroa83@knu.ac.kr (M.K.); pathpjy@naver.com (N.J.-Y.P.); 2 Department of Radiology, School of Medicine, Kyungpook National University, Daegu 41944, Republic of Korea; jmmpark@gmail.com (J.P.); blushain@naver.com (J.H.); 3 Department of Radiation Oncology, School of Medicine, Kyungpook National University, Daegu 41944, Republic of Korea; mkkang@knu.ac.kr; 4 Department of Internal Medicine, School of Medicine, Kyungpook National University, Daegu 41944, Republic of Korea; deblue00@naver.com (Y.R.L.); swjeon@knu.ac.kr (S.W.J.); 5 Department of Surgery, School of Medicine, Kyungpook National University, Daegu 41944, Republic of Korea; kwonhj95@naver.com

**Keywords:** hepatocellular carcinoma, extrahepatic metastasis, stomach, endoscopic biopsy, gastric polyp

## Abstract

**Background**: Although extrahepatic metastasis of hepatocellular carcinoma (HCC) is common in the lungs and bones, metachronous gastric metastasis of HCC, which is initially detected in the form of multiple small gastric polyps, is extremely rare. **Case presentation:** A 62-year-old man was diagnosed with HCC by abdominal computed tomography (CT), but the patient received only intermittent treatment. After 15 months, esophagogastroduodenoscopy revealed multiple 0.3–0.6 cm sessile polyps in the midbody and distal body of the stomach; these findings were not documented in the report of the previous examination. Microscopically, the stomach biopsy specimen revealed tumor cells with prominent nuclear atypia arranged in trabecular and solid patterns and infiltrating the gastric mucosa. However, no signs of glandular intraepithelial neoplasia were found in the gastric mucosa. Immunohistochemically, the tumor was positive for HepPar-1 and glypican-3 and negative for SALL4, cytokeratin-7, and cytokeratin-20, consistent with the immunophenotype of the primary HCC. Therefore, the gastric polyps were diagnosed as metachronous gastric metastasis of HCC. The patient was administered sorafenib, but follow-up CT showed radiological disease progression. He died 3 months after being diagnosed with metachronous gastric metastasis of HCC. **Conclusions**: Based on our experience, this rare case might help clinicians and pathologists avoid misdiagnosing gastric metastasis of HCC as primary gastric adenocarcinoma with hepatoid differentiation and prevent inappropriate treatment. In patients with HCC, biopsy of newly detected gastric polyps should be considered even when they appear benign on endoscopy, to exclude metastatic involvement.

## 1. Introduction

Primary liver cancer was the sixth most commonly diagnosed cancer and the third leading cause of cancer-related death worldwide in 2020 [1]. Hepatocellular carcinoma (HCC) is an etiology-driven cancer caused by chronic liver disease or exogenous exposure [2,3]. Chronic liver disease is caused by hepatitis B, hepatitis C, alcohol-associated and nonalcoholic steatohepatitis, and several inherited metabolic diseases affecting the liver. Extrahepatic dissemination from HCC has been observed in 30–75% of patients [4]. The lungs and bones are the most common sites of extrahepatic metastasis. However, gastrointestinal metastasis of HCC, particularly in the stomach, is extremely rare. When HCC involves the gastrointestinal tract, most cases result from direct contiguous invasion rather than hematogenous metastasis, and gastrointestinal bleeding is a common clinical presentation [5,6]. An important diagnostic consideration is primary gastric hepatoid adenocarcinoma, which exhibits hepatocyte-like morphology and may express alpha-fetoprotein (AFP) [7]. This tumor may also express glypican-3 (GPC-3) and present with liver metastases, potentially mimicking HCC with gastric metastasis [7,8,9]. These overlapping features can complicate the determination of the primary tumor site when both gastric and hepatic lesions are present, highlighting the importance of integrating clinical, radiological, and histopathological findings. Herein, we present a rare case of gastric metastasis from HCC incidentally detected as multiple small gastric polyps during esophagogastroduodenoscopy, illustrating this diagnostic challenge.

## 2. Case

**Figure 1:**
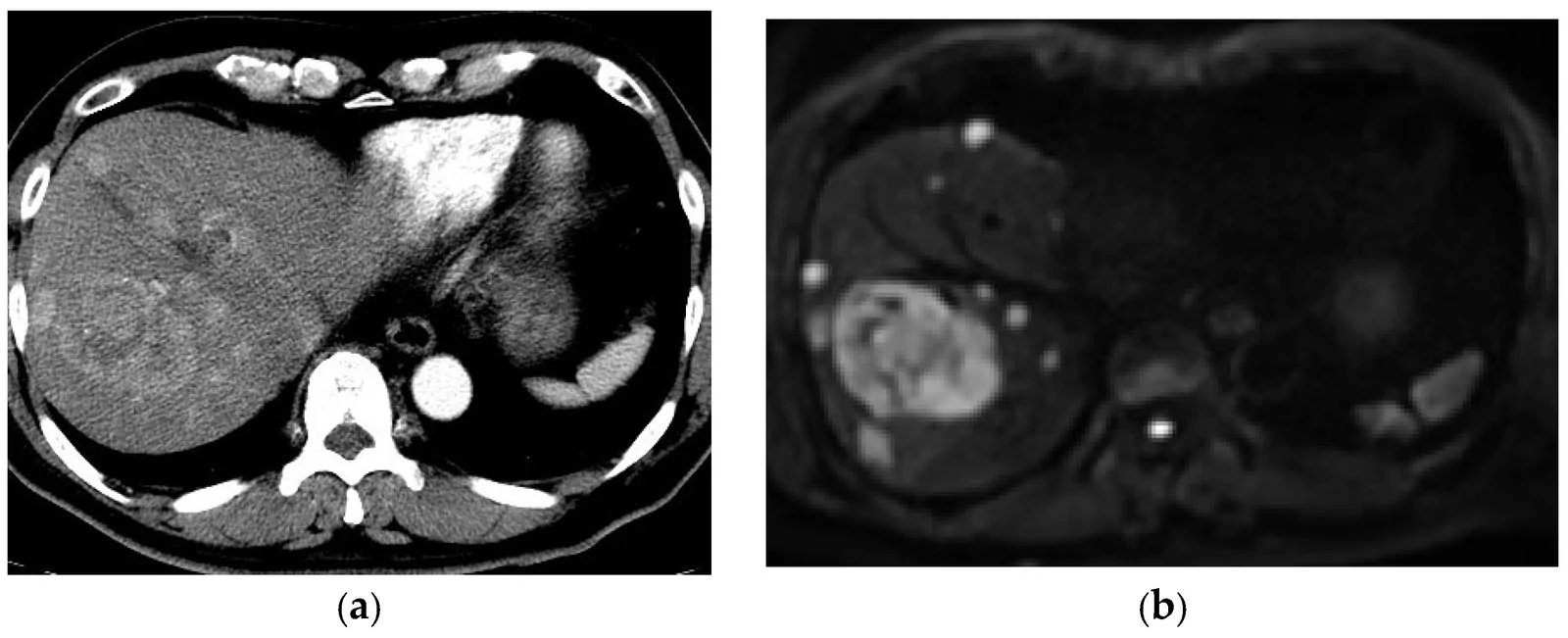
(**a**) Contrast-enhanced computed tomography of the abdomen and pelvis reveals multiple variable-sized hypervascular masses in bilateral lobes of the liver. (**b**) Diffusion-weighted magnetic resonance imaging of the liver shows diffusely distributed multinodular HCC involving both lobes with enlarged lymph nodes.

In August 2020, a 62-year-old Korean man presented to our hospital with a liver mass discovered incidentally during a routine health examination. He had been relatively healthy up until recently, with no clinical symptoms. He had a history of hypertension for several years, which had been treated with standard medications. During the preceding 5 years, he had consumed half a bottle to one bottle of soju (a Korean distilled alcoholic beverage containing approximately 18% alcohol by volume) per drinking occasion, three or more times per week. Initial contrast-enhanced computed tomography (CT) of the abdomen and pelvis revealed multiple variable-sized hypervascular masses throughout both lobes of the liver, the largest of which was 6.6 cm (Figure 1a). Other than in the liver, no abnormal findings suggestive of malignancy were observed. According to the written examination reports, esophagogastroduodenoscopy (EGD) and colonoscopy performed during the routine health examination revealed no unusual findings. Preliminary liver function tests showed elevated levels of aspartate aminotransferase (AST) (64 U/L; normal levels ≤ 37), alanine aminotransferase (ALT) (108 U/L; normal levels ≤ 41), total bilirubin (1.28 mg/dL; normal levels, 0.30–1.20), and gamma-glutamyl transferase (γ-GTP) (250 U/L; normal levels, 9–40). Serologic tests were positive for hepatitis B surface antigen (HBsAg) and immunoglobulin G antibody to hepatitis B core antigen, but negative for hepatitis B surface antibody (anti-HBs). The serum hepatitis B virus (HBV) DNA level at initial presentation was 363,000 IU/mL, as measured by real-time quantitative polymerase chain reaction. Antiviral therapy with Viread (300 mg orally once daily) was initiated at that time. Serum anti-hepatitis C virus antibody was also negative. Serum tumor marker testing revealed that the levels of AFP and protein induced by vitamin K absence or antagonist-II (PIVKA-II) were 6.8 ng/mL (normal level, ≤8.1 ng/mL) and 18,016.22 mAU/mL (normal level, 0–40 mAU/mL), respectively. Liver magnetic resonance imaging (MRI) revealed diffusely distributed multinodular HCC involving both lobes as well as enlarged lymph nodes (LNs) in the periportal region and along the common hepatic artery, raising the possibility of metastatic LNs (Figure 1b). Based on the radiologic findings, elevated PIVKA-II levels, and the patient’s underlying HBV infection, HCC was highly suspected, and an ultrasonography-guided biopsy of the liver mass was attempted using an 18-gauge needle (Stericut; TSK Laboratory, Tochigi, Japan). Histology of the needle biopsy specimen from the liver revealed moderately differentiated HCC. After diagnosis, the patient refused treatment for HCC and was then informed of the possibility of disease progression, but no special treatment was provided.

**Figure 2:**
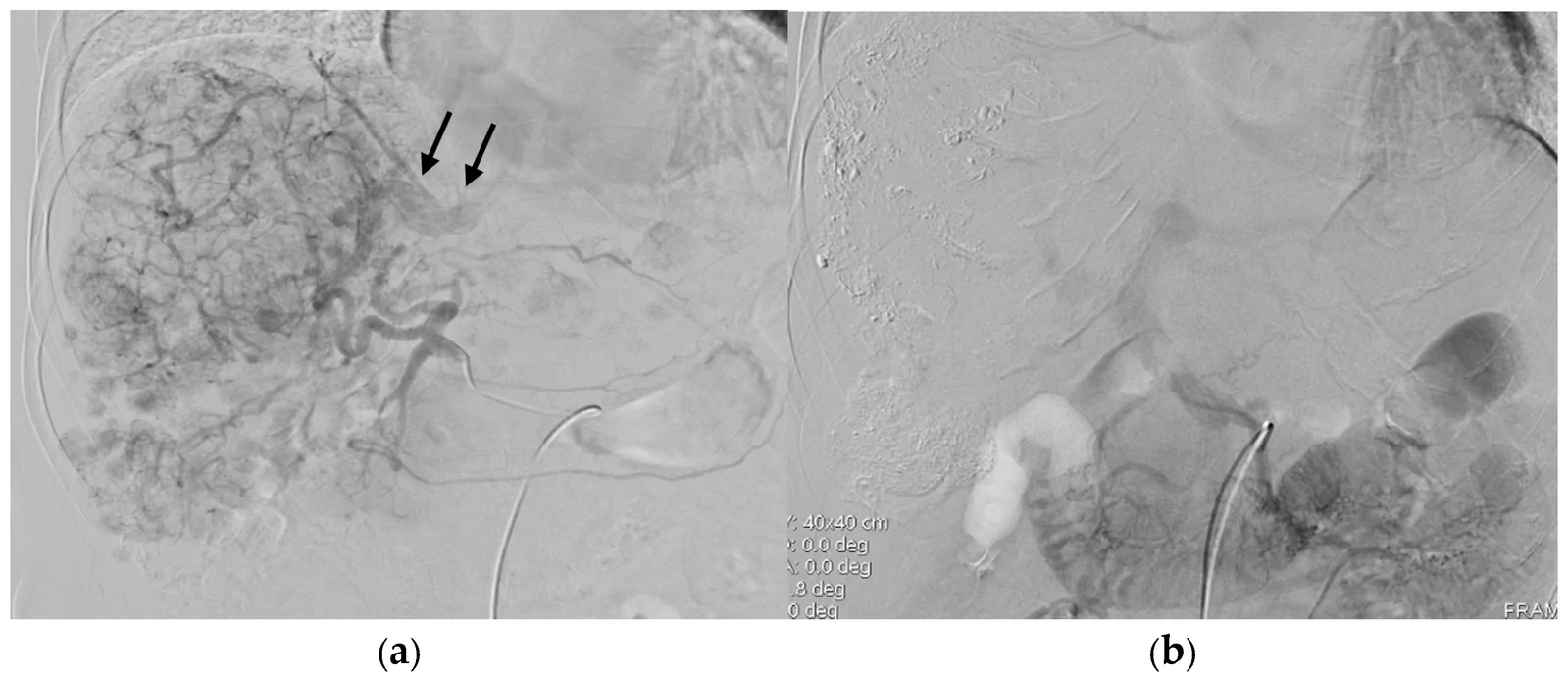
(**a**) Initial common hepatic angiography demonstrated multiple hypervascular masses and nodules in both hepatic lobes. An arteriovenous shunt (arrows), presumably communicating with the right hepatic vein, was also observed in association with a large hypervascular mass in the right hepatic lobe. No definite arterioportal shunt was identified on angiography. (**b**) Indirect portography obtained during the second TACE session demonstrated no evidence of hepatofugal portal venous flow or portal vein tumor thrombosis.

Four months after diagnosis, the patient underwent his first conventional transarterial chemoembolization (TACE) in December 2020. The procedure was performed using an emulsion of 10 mL of iodized oil (Lipiodol; Guerbet, Villepinte, France) and 50 mg of doxorubicin (Ildong Pharmaceutical Co., Ltd., Seoul, Republic of Korea), followed by embolization with absorbable gelatin sponge particles (Cali-Gel; Alicon Pharm SCI&TEC Co., Ltd., Hangzhou, China). Initial hepatic angiography demonstrated a hepatic artery–hepatic vein shunt, presumably associated with tumor invasion of the right hepatic vein (Figure 2a). However, no arterioportal shunt was identified on initial angiography. On the CT image taken after the first TACE, residual viable tumors were observed in many areas, with the largest measuring 10.2 cm along with resolving hemoperitoneum that suggested ruptured HCC. He also underwent a second TACE in February 2021. Indirect portography performed during this session showed no evidence of portal vein tumor thrombosis or hepatofugal portal flow (Figure 2b). Following the second TACE, CT revealed residual viable tumors in many areas as well as new nodules in the left adrenal gland (size: 1.2 cm) and right subhepatic area (size: 0.7 cm). A review of serial CT examinations demonstrated that mild splenomegaly and esophagogastric varices were already present prior to the initial TACE, without significant interval progression during follow-up. Furthermore, no convincing imaging evidence of aggravated portal hypertension was identified following TACE. Based on these imaging findings, the patient was treated with lenvatinib (Lenvima^®^, 4 mg, three capsules once daily) considering that he had stage IV disease in March 2021. To evaluate the patient’s response to lenvatinib, contrast-enhanced abdominal CT was performed on 27 May 2021, and showed a partial response according to the modified Response Evaluation Criteria in Solid Tumors (mRECIST). Although liver cirrhosis, splenomegaly, and esophageal varices were observed, no ascites was found.

**Figure 3:**
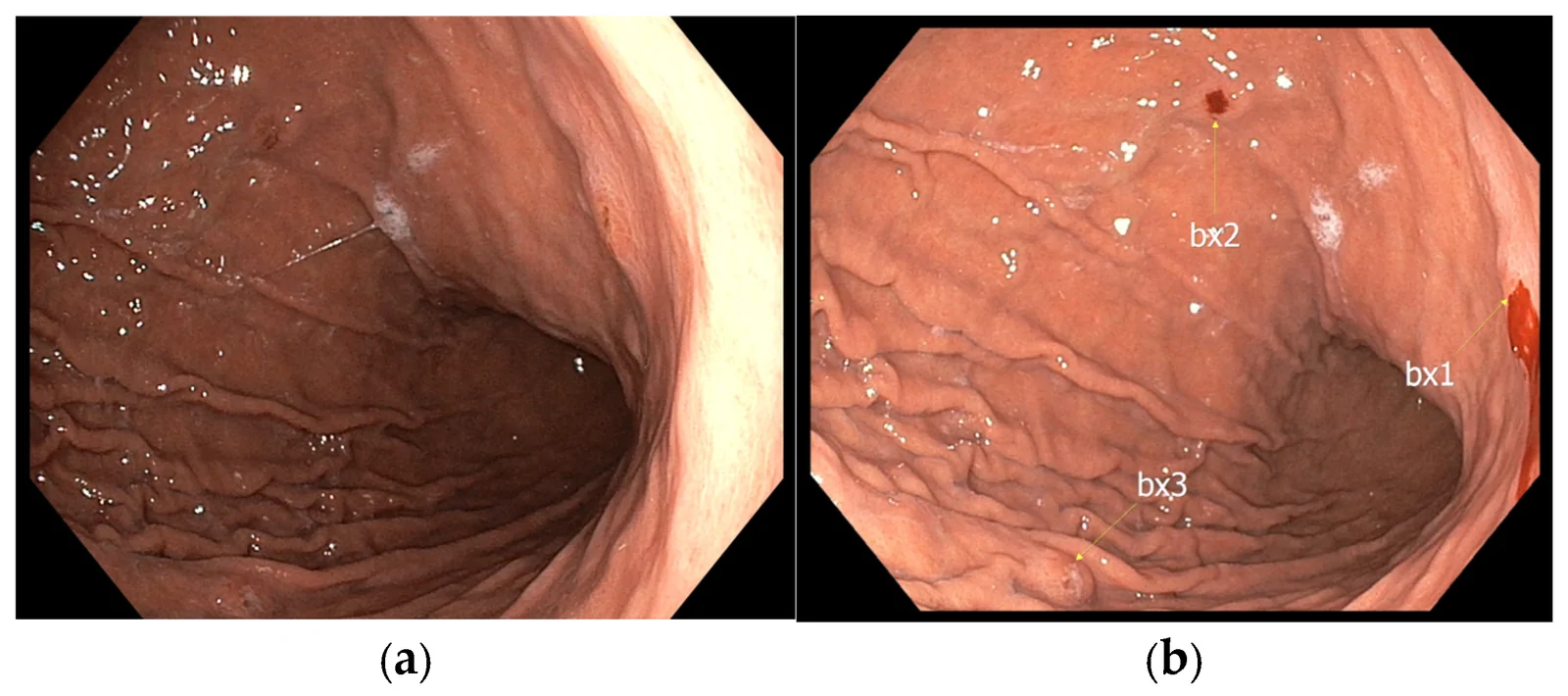
Representative image of esophagogastroduodenoscopy showing multiple sessile polyps. (**a**) The original image and (**b**) the same image with biopsy sites marked (bx: biopsy).

**Figure 4:**
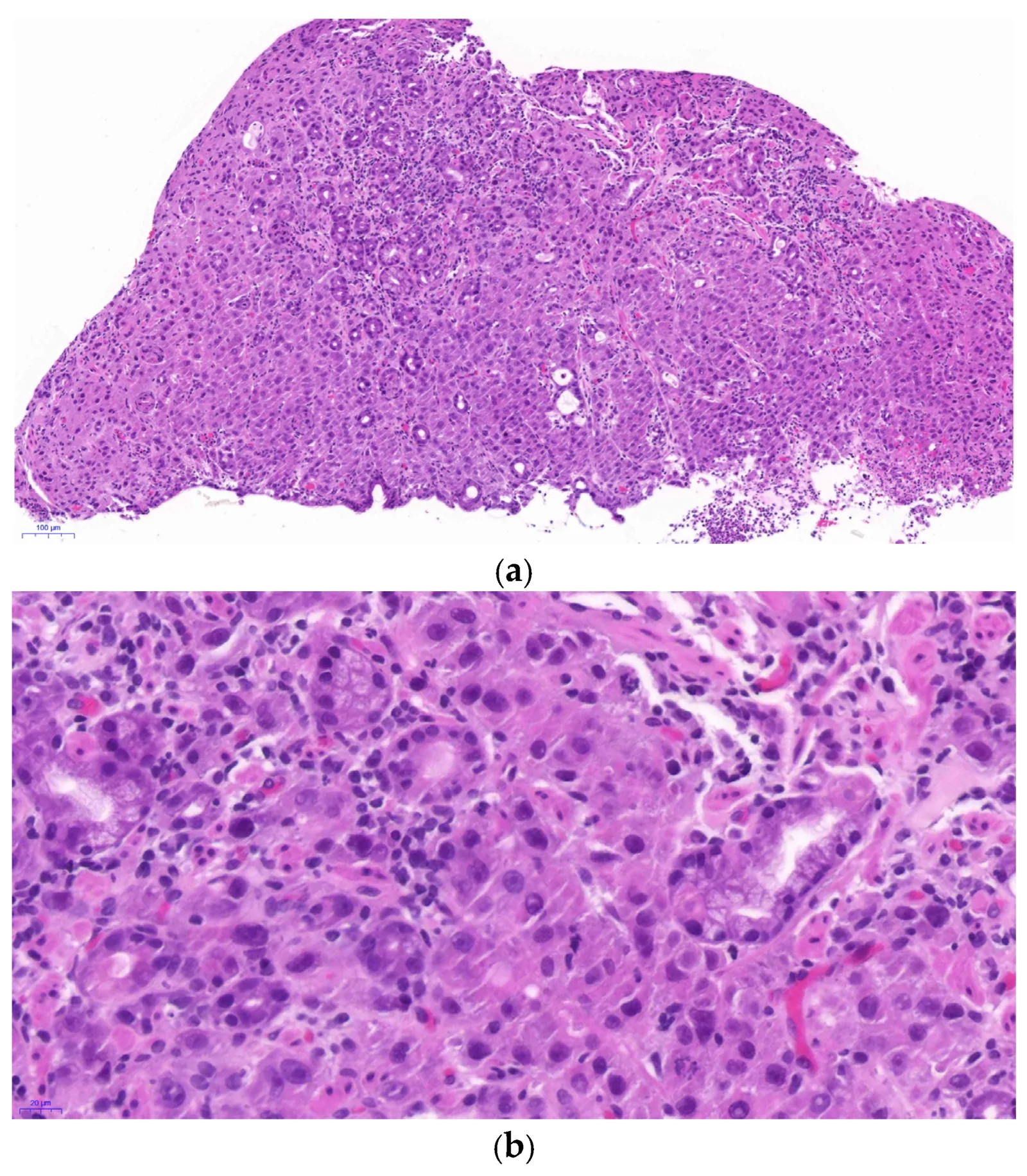
(**a**) Microscopically, tumor cells are arranged in trabecular and solid patterns, with marked nuclear atypia and infiltration of the gastric glands within the mucosa (10× objective). (**b**) Tumor cells show prominent nucleoli and mitosis (40× objective).

The patient had developed dyspepsia in the previous month and underwent EGD in November 2021. EGD revealed multiple 0.3–0.6 cm sessile polyps on the anterior wall and greater curvature of the midbody and on the posterior wall and lesser curvature of the distal body of the stomach (Figure 3), which had not been documented in the written report of a prior EGD performed at an outside hospital 15 months earlier. The images from that prior examination were unavailable for review. These polyps had a thin mucosa with an obvious underlying vascular pattern. Microscopic examination of a stomach biopsy specimen revealed tumor cells arranged in trabecular and solid patterns, with prominent nuclear atypia and infiltration of the gastric glands within the mucosa (Figure 4). However, no signs of glandular intraepithelial neoplasia were observed in the gastric mucosa.

**Figure 5:**
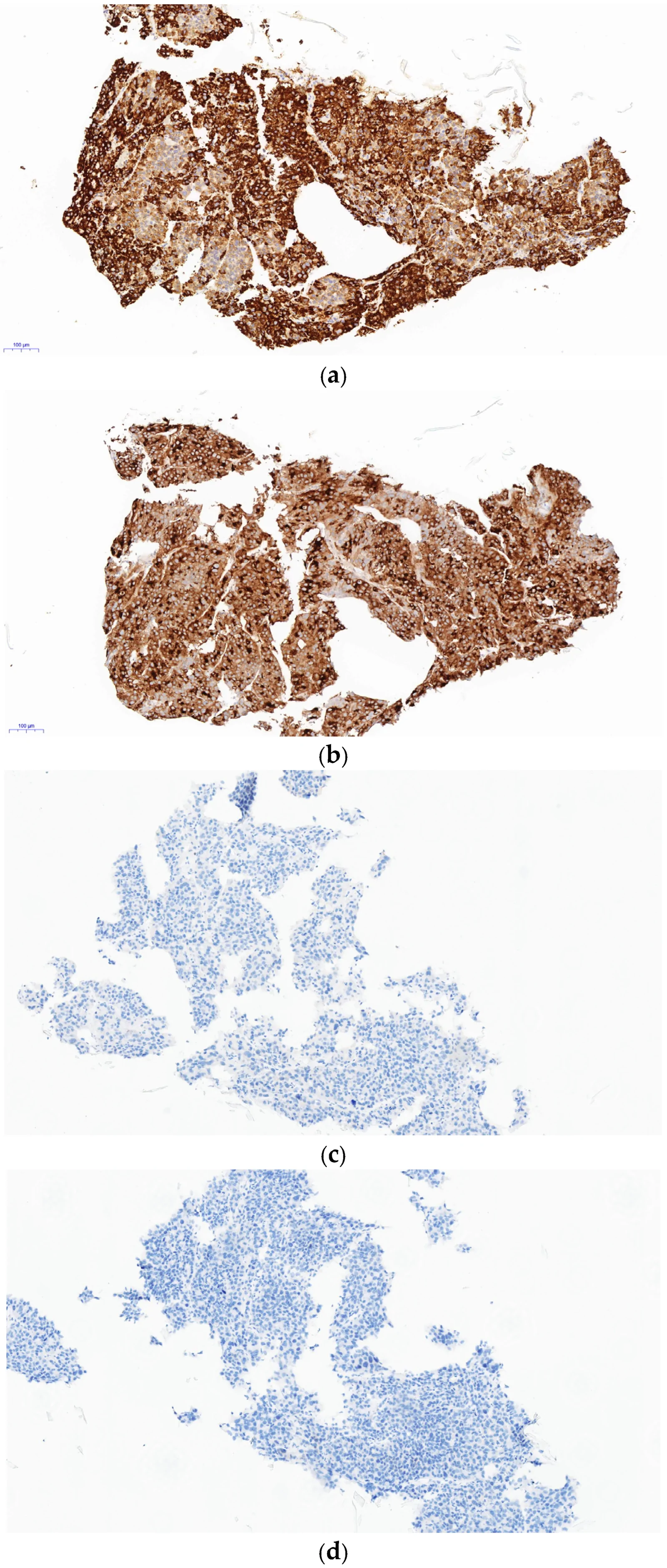

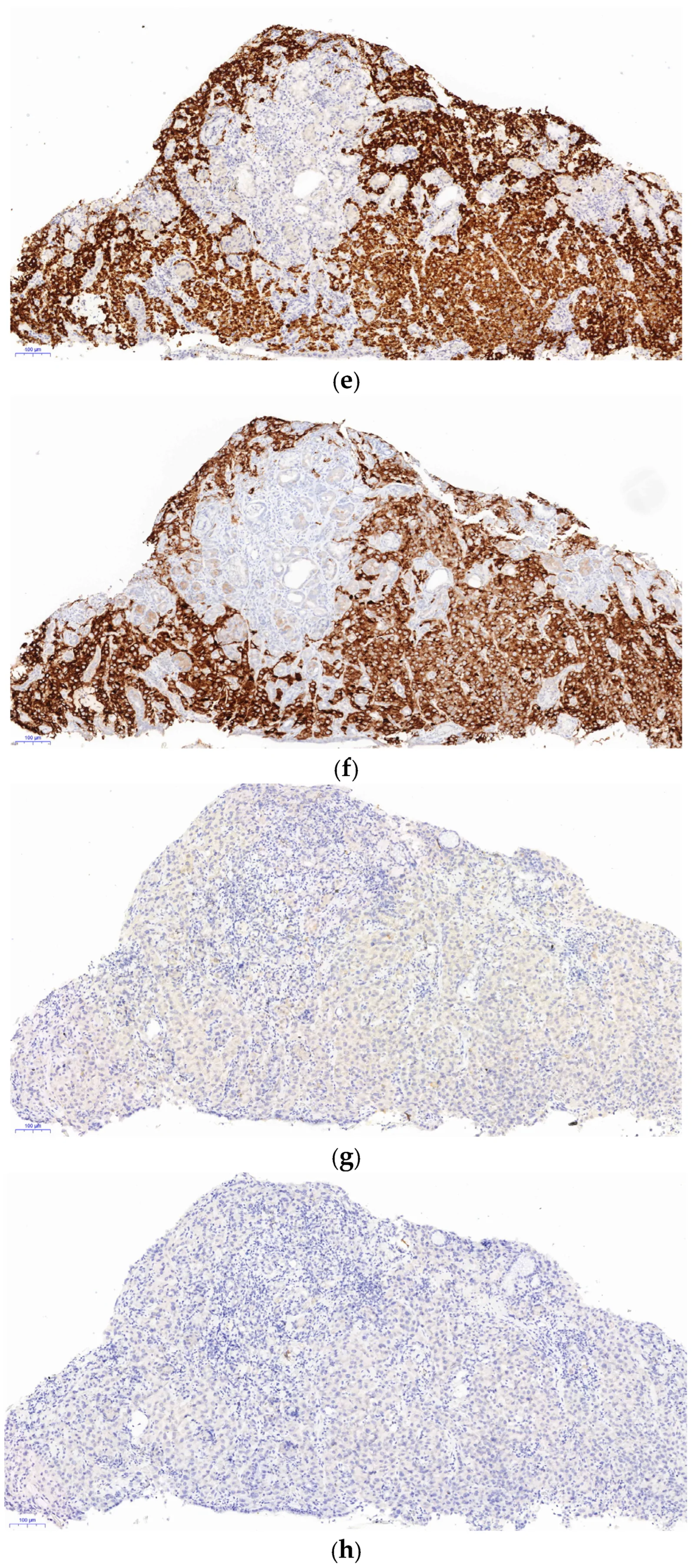
Immunohistochemical findings in hepatocellular carcinoma (HCC) and metachronous gastric metastasis of HCC. HCC tumor cells were diffusely and strongly positive for (**a**) HepPar-1 and (**b**) glypican-3 (GPC-3) but negative for (**c**) SALL4 and (**d**) AFP. Tumor cells from the stomach were found to be diffusely and strongly positive for (**e**) HepPar-1 and (**f**) GPC-3 but negative for (**g**) SALL4 and (**h**) AFP (10× objective).

Immunohistochemical (IHC) staining revealed positivity for HepPar-1 and GPC-3, but negativity for SALL4, AFP, cytokeratin (CK)7, and CK20 (Table 1), consistent with the immunophenotype of the primary tumor and confirming the diagnosis of metastatic HCC (Figure 5). Because CT showed no direct invasion by HCC and no thickening of the stomach wall or protruding mass lesion of the stomach, the gastric polyps were diagnosed as metachronous gastric metastasis of HCC.

Following the diagnosis of gastric metastasis in November 2021, disease progression was confirmed, and the patient was switched from lenvatinib to sorafenib (Nexavar^®^, 400 mg twice daily) in December 2021. Follow-up CT in January 2022 showed an increase in the size and number of enhancing lesions in both hepatic lobes, increased peripheral viable tumor within partially lipiodolized nodules in the right lobe, and enlargement of the left adrenal metastasis from 1.0 to 1.9 cm. These findings indicated radiological disease progression, although a formal mRECIST response category was not documented. The patient subsequently developed acute kidney injury, suspected to be secondary to dehydration or hepatorenal syndrome. His condition deteriorated with oliguria, progressive abdominal distension, and peripheral edema. Despite dual therapy with terlipressin and intravenous albumin, renal function continued to decline, and the patient died in February 2022, approximately 3 months after the diagnosis of gastric metastasis.

## 3. Discussion

HCC can exhibit capsular, extracapsular, and vascular invasion, as well as intrahepatic metastases [10]. HCC can also extend directly beyond the liver capsule or give rise to peritoneal metastases following tumor rupture. As mentioned earlier, extrahepatic metastases of HCC most commonly involve the lungs, abdominal LNs, and bones and are associated with poor prognosis [11,12,13]. However, gastrointestinal involvement by HCC is extremely rare. Most reported cases result from direct invasion and show endoscopic features resembling advanced carcinoma [14]. Hematogenous metastasis to the gastrointestinal tract has also been reported [15,16]. The presence of multiple small sessile polyps on EGD in our patient suggested hematogenous spread. Wu et al. reviewed the medical records of 48,473 patients with solid malignancies and identified gastric metastasis from HCC in only 6 patients (0.01%) [17]. In their study, gastric metastasis was detected after resection of the primary HCC in all six patients, with a mean interval of 33.8 months, and four of the six patients presented with melena [17]. In contrast, our patient presented with dyspepsia 15 months after the initial detection of primary HCC. Lin et al. reported gastric involvement in only 5 of 2237 patients with HCC (0.22%) [6]. The presumed route of spread was direct invasion in three patients, hematogenous spread in one, and undetermined in one [6]. Chen et al. reported gastric involvement in only 3 of 396 patients with HCC (0.76%) [5]. In the series reported by Lin et al., gastric involvement by HCC appeared endoscopically as submucosal tumors, an ulcerative tumor, or a penetrating ulcer [6]. Sakumura et al. described a gastric metastasis from HCC presenting as a small polyp with prominent vascular markings on the anterior wall of the gastric angle, with its margins becoming clearer after indigo carmine spraying [18]. Their case and ours illustrate that gastric metastasis from HCC can mimic early gastric cancer on EGD and that immunohistochemical staining can aid in establishing the correct diagnosis.

Several hypotheses have been proposed to explain gastrointestinal involvement by HCC, including (1) direct invasion of the gastrointestinal tract after TACE and/or transcatheter arterial embolization (TAE), (2) hematogenous spread of tumor thrombi through the portal venous system, and (3) intraperitoneal implantation [5,19,20]. Our patient underwent two TACE sessions before EGD was performed for dyspepsia. However, this temporal association does not establish a causal relationship between TACE and gastric metastasis. Gastric metastasis may instead reflect the natural progression of the patient’s advanced multifocal HCC. The possibility that TACE-related changes in portal venous pressure contributed to hematogenous spread remains speculative, as there is no direct evidence supporting this mechanism in our patient.

Notably, hepatoid adenocarcinoma of the stomach resembles HCC morphologically. This tumor may produce AFP and show immunoreactivity for AFP by IHC. Gastric hepatoid adenocarcinoma is characterized by older age and an aggressive clinical course, which includes vascular invasion and liver metastasis [9,21]. As a result, liver metastasis of gastric hepatoid adenocarcinoma can be misdiagnosed as HCC. Furthermore, GPC-3 is expressed in hepatoid, clear-cell, and AFP-producing gastric cancer, yolk sac tumors, and HCC [8]. Therefore, when synchronous masses are found in the liver and stomach, it is necessary to differentiate between gastric hepatoid adenocarcinoma and HCC, even in patients with underlying hepatitis B virus infection. SALL4 can be used as a diagnostic IHC marker to distinguish gastric hepatoid adenocarcinoma from HCC [21]. In a comparative study between 338 gastric adenocarcinomas and 60 HCCs, SALL4 showed completely negative expression in 60 HCCs [21]. However, SALL4 expression has also been reported in a progenitor-like subtype of HCC associated with an aggressive phenotype [22]. In our case, the AFP level was within normal limits, whereas the PIVKA-II level was significantly elevated, and HCC was diagnosed before any gastric lesion was documented. In addition, both the liver and gastric specimens were negative for AFP and SALL4. Based on these findings, we concluded that the patient had metachronous gastric metastasis of HCC. However, clinicians performing EGD should consider a wide range of differential diagnoses because stomach cancer can present as small gastric polyps.

In summary, we presented a case of metachronous gastric metastasis of HCC discovered incidentally during esophagogastroduodenoscopy, which revealed multiple small gastric polyps.

## 4. Conclusions

Based on our experience, this rare case might help clinicians and pathologists avoid misdiagnosing gastric metastasis of HCC as primary gastric adenocarcinoma with hepatoid differentiation and prevent inappropriate treatment. In patients with HCC, biopsy of newly detected gastric polyps should be considered even when they appear benign on endoscopy, to exclude metastatic involvement.

## Figures and Tables

**Table 1:** Panel of primary antibodies used for immunohistochemical analysis *.

Antibody	Clone	Dilution	Manufacturer
**HepPar-1**	OCH1E5	1:100	Dako/Agilent Technologies, Glostrup, Denmark
**Glypican-3**	GC33	Ready-to-use	Ventana Medical Systems, Tucson, AZ, USA
**SALL4**	6E3	1:100	Cell Marque, Rocklin, CA, USA
**AFP**	Polyclonal	1:2	Dako/Agilent Technologies, Glostrup, Denmark
**Cytokeratin 7**	OV-TL 12/30	1:400	Dako/Agilent Technologies, Glostrup, Denmark
**Cytokeratin 20**	Ks20.8	1:4	Dako/Agilent Technologies, Glostrup, Denmark

* Immunohistochemical staining was performed on 4-µm-thick formalin-fixed, paraffin-embedded tissue sections using an automated slide stainer (BenchMark ULTRA; Ventana Medical Systems, Tucson, AZ, USA). Heat-induced epitope retrieval was achieved with Cell Conditioning 1 (CC1) buffer (Ventana Medical Systems). Bound primary antibodies were detected using the ultraView Universal DAB Detection Kit (Ventana Medical Systems). Sections were subsequently counterstained with Hematoxylin II, followed by treatment with Bluing Reagent (both from Ventana Medical Systems).

## Data Availability

The datasets generated or analyzed during the study are available from the corresponding author on reasonable request.

## Abbreviations

The following abbreviations are used in this manuscript:

HCC	Hepatocellular carcinoma
AFP	Alpha-fetoprotein
GPC-3	Glypican-3
CT	Computed tomography
MRI	Magnetic resonance imaging
EGD	Esophagogastroduodenoscopy
AST	Aspartate aminotransferase
ALT	Alanine aminotransferase
γ-GTP	Gamma-glutamyl transferase
HBsAg	Hepatitis B surface antigen
HBV	Hepatitis B virus
PIVKA-II	Protein induced by vitamin K absence or antagonist-II
LN	Lymph node
TACE	Transarterial chemoembolization
mRECIST	Modified Response Evaluation Criteria in Solid Tumors
IHC	Immunohistochemistry
HepPar-1	Hepatocyte paraffin 1
CK	Cytokeratin
TAE	Transcatheter arterial embolization
